# Integrative and complementary practices in diabetes management: a rapid review of clinical guidelines

**DOI:** 10.1590/S2237-96222026v35e20250602.en

**Published:** 2026-03-04

**Authors:** Marcus Tolentino Silva, Daniel Miele Amado, Paulo Roberto Sousa Rocha, Jorge Otávio Maia Barreto

**Affiliations:** ¹ Universidade de Brasília, Departamento de Saúde Coletiva, Brasília, DF, Brazil Universidade de Brasília Departamento de Saúde Coletiva Brasília DF Brazil; ² Ministério da Saúde, Núcleo Técnico de Gestão da Política Nacional de Práticas Integrativas e Complementares em Saúde no Sistema Único de Saúde, Brasília, DF, Brazil Ministério da Saúde Núcleo Técnico de Gestão da Política Nacional de Práticas Integrativas e Complementares em Saúde no Sistema Único de Saúde Brasília DF Brazil; ³ Fundação Oswaldo Cruz, Escola de Governo, Brasília, DF, Brazil Fundação Oswaldo Cruz Escola de Governo Brasília DF Brazil

**Keywords:** Practice Guideline, Complementary Therapies, Diabetes Mellitus, Evidence-Informed Policy, Rapid Review., Guía de Práctica Clínica, Terapias Complementarias, Diabetes Mellitus, Política Informada por la Evidencia, Revisión Rápida

## Abstract

**Objective:**

To map clinical guidelines that used the Grading of Recommendations, Assessment, Development and Evaluation (GRADE) system to make recommendations on integrative and complementary health practices (ICHP) in diabetes management.

**Methods:**

The search was conducted in Medline/PubMed, Embase, and Scopus on 24 October 2024, with no date limits. Guidelines that applied the GRADE system were included. Title and abstract screening was duplicated and performed independently. One reviewer conducted full-text reading and data extraction, and another performed verification. Methodological quality was assessed using the Appraisal of Guidelines for Research and Evaluation II (AGREE II) instrument, specifically the rigor of the development domain. A narrative synthesis was organized by outcome.

**Results:**

Of the seven guidelines included, five showed high methodological confidence, one moderate, and one low. Recommendations were favorable for psychological support, self-care education, foot- and ankle-focused exercises, low-impact exercises, mindfulness, Ayurveda, and essential oils as additional supports; they were unfavorable for apitherapy and ozone therapy. Heterogeneity in outcomes and variation in the consistency of recommendations were observed.

**Conclusion:**

There are insufficient guidelines on ICHP in diabetes management, with methodological variability and inconsistent recommendations. New guidelines, with greater rigor and transparency, may support decision-making regarding the incorporation of ICHP into the Brazilian Unified Health System (Sistema Único de Saúde, SUS). Among the practices assessed, exercise, mindfulness, and self-care showed stronger evidence and may be prioritized in primary care for diabetes management.

## Resumen

**Objetivo:**

Mapear guías clínicas que utilizaron el sistema de Clasificación de la Evaluación, el Desarrollo y la Evaluación de Recomendaciones (Grading of Recommendations, Assessment, Development and Evaluation, GRADE) para emitir recomendaciones sobre prácticas integrativas y complementarias en salud (PICS) en el manejo de la diabetes.

**Métodos:**

La búsqueda se realizó en Medline/PubMed, Embase y Scopus el 24 de octubre de 2024, sin límite de fecha. Se incluyeron guías que aplicaron el sistema GRADE. La selección de títulos y resúmenes fue duplicada e independiente. Un revisor realizó la lectura completa y la extracción de datos, y otro efectuó la verificación. La calidad metodológica se evaluó mediante el Instrumento de Evaluación de Guías para la Investigación y Evaluación II (Appraisal of Guidelines for Research and Evaluation II, AGREE II), en el dominio de rigor en el desarrollo. Se organizó una síntesis narrativa por desenlace.

**Resultados:**

De las siete guías incluidas, cinco presentaron alta confianza metodológica, una moderada y otra baja. Las recomendaciones fueron favorables para el apoyo psicológico, la educación en autocuidado, los ejercicios dirigidos al pie y al tobillo, los ejercicios de bajo impacto, la atención plena, el ayurveda y los aceites esenciales como soporte adicional, y desfavorables para la apiterapia y la ozonoterapia. Se observó heterogeneidad en los desenlaces y variación en la consistencia de las recomendaciones.

**Conclusión:**

Existe escasez de guías sobre PICS en el manejo de la diabetes, con variabilidad metodológica y recomendaciones inconsistentes. Nuevas guías, con mayor rigor y transparencia, pueden apoyar la toma de decisiones sobre la incorporación de PICS al Sistema Único de Salud (Sistema Único de Saúde, SUS). Entre las prácticas evaluadas, los ejercicios, el mindfulness y los componentes de autocuidado presentaron evidencia más prometedora, lo que permite priorizarlos en la atención primaria del cuidado de la diabetes.

**Ethical aspects**

This research used public domain anonymized databases.

## Introduction

Integrative and complementary health practices (ICHP) encompass mind-body interventions, biological therapies, and traditional medical systems. In Brazil, their regulation is formalized through the National Policy on Integrative and Complementary Practices (Política Nacional de Práticas Integrativas e Complementares, PNPIC), in force since 2006 within the Brazilian Unified Health System (Sistema Único de Saúde, SUS), which provides for the offering of modalities such as acupuncture, phytotherapy, meditation, and yoga. ^1^.

Diabetes represents one of the chronic conditions with the greatest global impact, with 529 million people affected in 2021 and a projected increase to more than 1.31 billion cases by 2050 ^2^. Type 2 diabetes is estimated to have accounted for 97% of diabetes-related deaths between 1990 and 2021, reflecting the burden of the disease in terms of mortality and disability ^3^. In Brazil, data from 2019 indicated a self-reported prevalence of 8% (95% confidence interval: 7.4; 8.0) among adults, confirming the magnitude of the problem at the national level ^4^.

The Grading of Recommendations, Assessment, Development and Evaluation (GRADE) system is widely recommended as the standard approach for assessing health evidence ^5^^,^^6^, but its adoption in clinical guidelines remains inconsistent. The method classifies the quality of evidence (high, moderate, low, or very low) and the strength of recommendations (weak, conditional, or strong), providing greater transparency to the process of formulating clinical recommendations. In Australia, between 2011 and 2018, only 15 of the 240 guideline documents assessed applied GRADE, and its application was heterogeneous ^7^. This variability reinforces the importance of critically analyzing how recommendations on ICHP have been formulated.

Although conventional diabetes management is well established, persistent challenges related to self-care, treatment adherence, and the psychosocial impact of the disease have driven growing interest in ICHP and their expansion within the SUS through the National Policy on Integrative and Complementary Practices (PNPIC). Scaling up these interventions requires consistent clinical guidelines to inform decisions about the benefits, risks, and uncertainties of these practices in the care of people with diabetes. Therefore, there is a clear need to analyze existing recommendations, particularly in light of GRADE, to support managers and health professionals in their decision-making and to identify gaps that require more robust research.

Accordingly, this rapid review aimed to map clinical guidelines that used the Grading of Recommendations, Assessment, Development and Evaluation (GRADE) system to make recommendations on integrative and complementary health practices (ICHP) in diabetes management.

## Methods

Protocol and registration

This rapid review was conducted in accordance with methodological guidelines developed from a Delphi panel with experts in health technology assessment ^8^. In this case, the method was chosen to address the need to provide timely evidence on ICHP within the SUS. The simplifications included a precise formulation of the research question, a focus on secondary studies, the use of specific appraisal tools, and data extraction by one reviewer with verification by another. 

The guiding question was structured using the PICO acronym (population, intervention, comparison, outcome) as follows.

Population: people with diabetes. 

Intervention: integrative and complementary practices in health. 

Comparison: not applicable. 

Outcome: glycemic control, wound healing, and prevention of ulcers and amputations. 

Reporting was adapted from the recommendations of the Preferred Reporting Items for Systematic Reviews and Meta-Analyses (PRISMA 2020) checklist ^9^. The protocol was previously registered on the Open Science Framework platform (available at: https://osf.io/q3sbw).

Eligibility criteria

Clinical guidelines that used the GRADE system to classify the quality of evidence and the strength of recommendations were included, to ensure comparability across documents and transparency in the assessment of recommendations ^10^. The guidelines were required to present explicit recommendations on the use of ICHP in people with type 1 diabetes, type 2 diabetes, or associated complications. There were no restrictions regarding language, country, or publication period.

The practices considered were those directly assessed in the included guidelines, encompassing interventions aimed not only at disease treatment but also at preventing complications, promoting self-care, and providing psychosocial support. Whenever possible, alignment with practices currently implemented within the SUS was sought. Guidelines that did not address ICHP, documents that did not use the GRADE system, expert consensus statements not based on a literature review, and guidelines without explicit methodology for evidence searching and synthesis were excluded.

Information sources

The databases Medline (via PubMed, Medical Literature Analysis and Retrieval System Online), Embase (Excerpta Medica Database), and Scopus (Elsevier abstract and citation database) were used. No searches were conducted in specialized guideline portals, such as the Guideline International Network or the National Institute for Health and Care Excellence; regional databases (e.g., Asian databases); or the gray literature, as this review was conducted in a rapid format, which required simplification of the search stage ^11^.

Search strategy

The search strategy was developed by MTS and validated by JOMB. The search was conducted on 24 October 2024, with no restrictions on language, country, or publication period. Terms related to "diabetes," "clinical guidelines," and the "GRADE system" were combined, with adjustments according to the specificity of each database.

Boolean operators and controlled descriptors (MeSH and Emtree) were used to balance sensitivity and specificity. Although the research question was structured according to the PICO acronym, interventions and outcomes were excluded from the strategy to maximize sensitivity and avoid missing potentially relevant guidelines (Table 1). 

**Table 1:** Search strategy in the selected information sources and results obtained on 24 October 2024

Source	Search terms	Results
Medline (via PubMed)	#1 “diabetes mellitus”[Mesh] OR (diabetes mellitus) OR (hyperglycemia AND glucose AND intolerance) #2 (guidelines as topic[MeSH:noexp] OR practice guidelines as topic[MeSH:noexp] OR Health Planning Guidelines[MeSH:noexp] OR practice guideline[MeSH:noexp] OR clinical protocols[MeSH:noexp] OR Consensus[MeSH:noexp] OR “Consensus Development Conference”[PTYP] OR “Consensus Development Conference, NIH”[PTYP] OR “Consensus Development Conferences as Topic”[MeSH:noexp] OR “Consensus Development Conferences, NIH as Topic”[MeSH:noexp] OR critical pathway[MeSH:noexp] OR (clinical[TIAB] AND pathway[TIAB]) OR (clinical[TIAB] AND pathways[TIAB]) OR (practice[TIAB] AND parameter[TIAB]) OR (practice[TIAB] AND parameters[TIAB]) OR algorithms[MeSH:noexp] OR care pathway[TIAB] OR care pathways[TIAB] OR guidance[TIAB] OR guideline*[TI]) #3 “GRADE system”[tiab] OR “GRADE methods”[tiab] OR “GRADE approaches”[tiab] OR “GRADE approach”[tiab] OR “Grading of Recommendations, Assessment, Development and Evaluation”[tiab] OR (“GRADE”[tiab] AND (“low”[tiab] OR “moderate”[tiab]OR “high”[tiab] OR “evidence”[tiab] OR “strong”[tiab] OR “weak”[tiab] “recommendation”[tiab])) #4 #1 AND #2 AND #3	119
Embase	1 ‘diabetes mellitus’/exp OR ‘diabetes mellitus’ OR ((‘hyperglycemia’/exp OR ‘hyperglycemia’) AND (‘glucose’/exp OR ‘glucose’) AND (‘intolerance’/exp OR ‘intolerance’)) #2 ‘practice guideline’/exp OR ‘practice guideline’ #3 ‘GRADE approach’/exp OR ‘GRADE approach’ #4 #1 AND #2 AND #3	104
SCOPUS	TITLE-ABS-KEY(“diabetes mellitus”) OR TITLE-ABS-KEY(“hyperglycemia” AND “glucose” AND “intolerance”)) AND (TITLE-ABS-KEY(“GRADE system” OR “GRADE methods” OR “GRADE approaches” OR “GRADE approach” OR “Grading of Recommendations, Assessment, Development and Evaluation”))	309

Screening process

The selection of studies took place in two stages. Initially, two independent reviewers (MTS and JOMB) screened titles and abstracts for eligibility. Subsequently, the full texts of potentially relevant records were assessed by MTS, with verification by JOMB. 

Discrepancies were resolved by consensus. Duplicate removal and screening organization were performed using EndNote X8.2 (Clarivate Analytics). During the drafting of the rapid review protocol, a pilot test was conducted to identify sentinel references and to enable adjustments and corrections to the search strategy.

Data extraction

Data extraction was performed by MTS using a standardized Microsoft Word spreadsheet and independently verified by JOMB. In cases of disagreement, decisions were reached by consensus among the reviewers. When a given variable was not clearly described in the documents, the expression "not reported" was used in the tables, without adding additional data or contacting guideline authors, in accordance with the rapid review design.

The following variables were extracted: guideline author and year, context (country or scope), target condition (type 1 diabetes, type 2 diabetes, or associated complications), integrative practices included, outcomes assessed, recommendations formulated, quality of evidence (very low, low, moderate, or high, according to GRADE), and strength of recommendation (weak, conditional, or strong, according to GRADE).

Methodological quality assessment

The methodological quality of the guidelines was assessed based on domain 3 (rigor of development) of the Appraisal of Guidelines for Research and Evaluation II (AGREE II) instrument ^12^. Only the eight items of this domain were used, rather than the full 28-item tool, considering the rapid review design and the focus on the robustness of recommendation formulation.

For each domain item, judgments were assigned as follows: yes (criterion met), partial (criterion partially met), or no (criterion not met). The items assessed were: systematic evidence search; explicit selection criteria; description of strengths and limitations; method for formulating recommendations; consideration of benefits and risks; transparent link between evidence and recommendations; external review; and provision for updating. The final classification of methodological confidence in the guidelines was defined as: high (all eight items met), moderate (some items partially met), and low (multiple "no" responses).

Data synthesis

The extracted information was synthesized into a narrative and presented in structured tables by clinical outcomes and the integrative practices evaluated. This presentation aimed to facilitate an integrated view of guideline methodological quality, consistency of recommendations, and the strength with which recommendations were issued.

Recommendation consistency was assessed by comparing the included guidelines for each practice and outcome, thereby identifying convergence, divergence, and the absence of recommendations. Recommendation strength was assessed using the GRADE system and classified as strong, weak, or conditional. The combination of recommendations was qualitative and aimed to achieve balance through critical analysis of the body of evidence.

## Results

The search identified 532 records. After removing 151 duplicates, 381 records remained for title and abstract screening. Of these, 326 were excluded for not meeting the eligibility criteria. Fifty-five full texts were assessed, with 48 guidelines excluded for not addressing ICHP. A detailed list of these guidelines is provided as supplementary material (available at: https://osf.io/q3sbw). Ultimately, seven clinical guidelines ^13^^-^^19^ were included in the synthesis (Figure 1).

**Figure 1:**
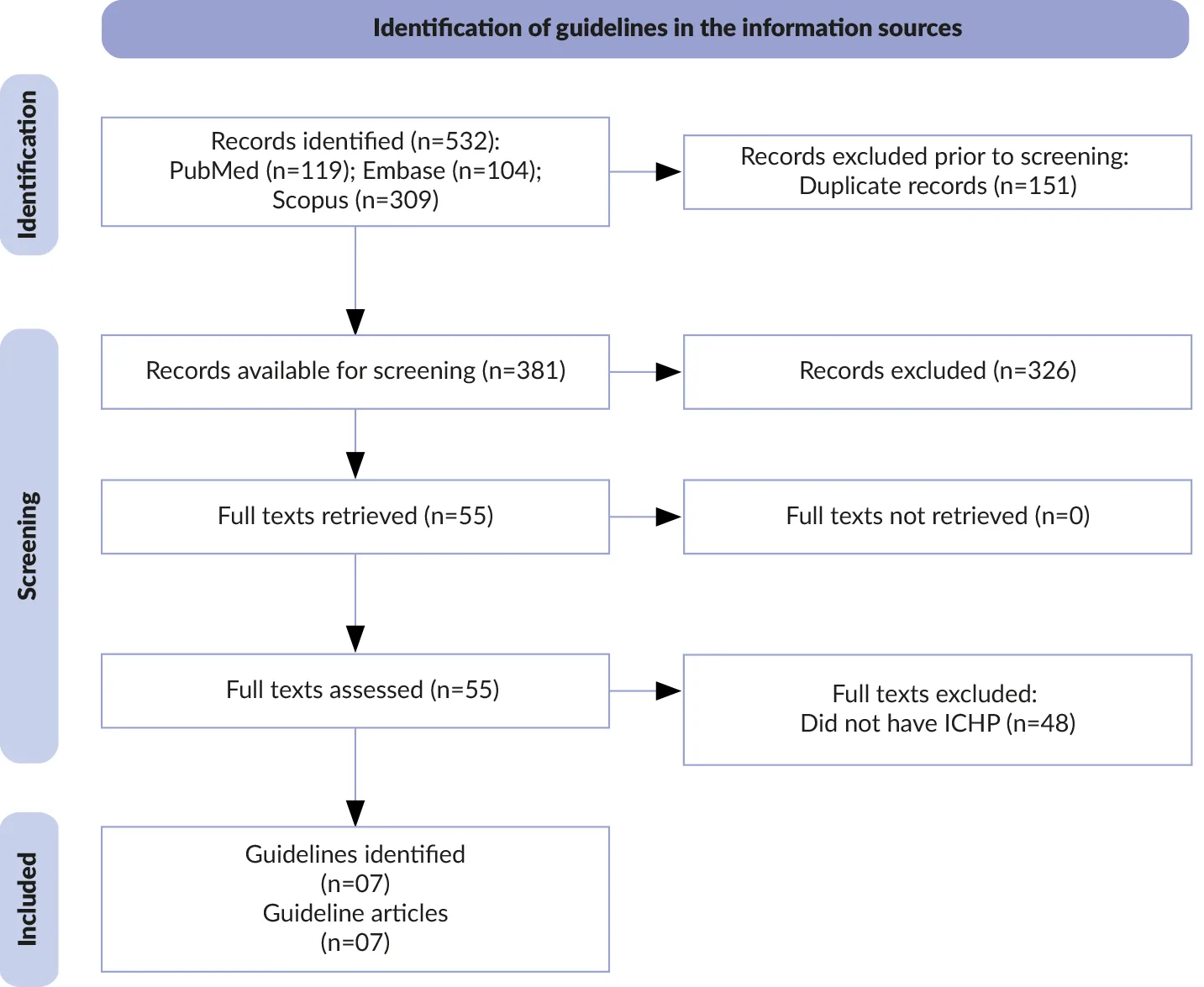
Clinical guidelines identification flow

The included guidelines were published between 2022 and 2024 and encompassed various global initiatives and contexts, including India, Australia, and the United States. The included guidelines addressed the management of type 2 diabetes and its complications, including diabetic foot ulcers and diabetes-associated infections (Table 2). Among the practices evaluated, notable interventions included foot- and ankle-focused exercises, self-care education, psychological support, mindfulness, Ayurveda, essential oils, low-impact exercises, apitherapy, and ozone therapy. The outcomes analyzed included glycemic control, ulcer prevention and healing, and the incidence of amputations.

Methodological assessment using the AGREE II instrument showed five guidelines with high confidence, one with moderate confidence, and one with low confidence (Table 3). Guidelines classified as high confidence consistently met criteria such as a systematic evidence search, a clear description of selection methods, a clear rationale for recommendation formulation, and an explicit link between recommendations and evidence. The guideline with moderate confidence partially met these criteria, while the guideline of the lowest quality did not meet any of the evaluated items. Final classifications were assigned according to the proportion of criteria met.

Recommendations across the guidelines showed variability (Table 4). Psychological support, self-care education, and foot- and ankle-focused exercises received conditional recommendations for preventing diabetic foot ulcers (moderate-quality evidence; high consistency). Ayurveda was conditionally recommended as an adjunct for glycemic control (moderate-quality evidence; high consistency). Low-impact exercises and mindfulness received weak recommendations for glycemic control (low-quality evidence; high consistency). Essential oils were suggested as additional support in diabetic foot management (low-quality evidence; low consistency). Apitherapy and ozone therapy were not recommended for ulcer healing or amputation prevention (low-quality evidence; consistency ranging from low to high).

**Table 2:** Characteristics of the included clinical guidelines on integrative and complementary health practices in diabetes management (n=7)

Autor, ano	Contexto da diretriz	Condição-alvo	Práticas avaliadas	Desfechos
Bus, 2023 ^13^	Global	Úlceras do pé diabético	Exercícios direcionados ao pé e tornozelo, educação em autocuidado, apoio psicológico	Prevenção de úlceras do pé diabético
Chattopadhyay, 2023 ^14^	Índia	Diabetes tipo 2	Ayurveda	Controle glicêmico
Chen, 2022 ^15^	Austrália	Úlceras do pé diabético	Ozonioterapia	Cicatrização de úlceras do pé diabético
Chen, 2024 ^16^	Global	Úlceras do pé diabético	Ozonioterapia	Cicatrização de úlceras do pé diabético
Commons, 2022 ^17^	Austrália	Infecções associadas ao diabetes	Apiterapia, óleos essenciais	Incidência de amputações
Conlin, 2024 ^18^	Estados Unidos	Diabetes tipo 2	Exercícios de baixo impacto, atenção plena	Controle glicêmico
Lazzarini, 2023 ^19^	Austrália	Úlceras do pé diabético	Apiterapia, ozonioterapia	Incidência de amputações

**Table 3:** Critical appraisal of the included clinical guidelines on diabetes according to the criteria of the Appraisal of Guidelines for Research and Evaluation II (AGREE II) instrument

Author, year	Systematic search	Screening process	Strengths/limitations described	Recommendation formulation	Benefits and risks considered	Link between recommendations and evidence	External review	Planned update	Guideline confidence
Bus, 2023 ^13^	Yes	Yes	Yes	Yes	Yes	Yes	Yes	Yes	High
Chattopadhyay, 2023 ^14^	Yes	Yes	Yes	Yes	Yes	Yes	Yes	Yes	High
Chen, 2022 ^15^	Partial	Partial	Partial	Partial	Yes	Partial	No	No	Moderate
Chen, 2024 ^16^	Yes	Yes	Yes	Yes	Yes	Yes	Yes	Yes	High
Commons, 2022 ^17^	No	No	No	No	No	No	No	No	Low
Conlin, 2024 ^18^	Yes	Yes	Yes	Yes	Yes	Yes	Yes	Yes	High
Lazzarini, 2023 ^19^	Yes	Yes	Yes	Yes	Yes	Yes	Yes	Yes	High

**Table 4:** Summary of recommendations for the use of integrative and complementary health practices in clinical guidelines on diabetes

Practice evaluated (reference)	Outcome	Targeting	Quality of evidence	Strength of recommendation	Consistency of recommendation
Apitherapy ^17^^,^^19^	Diabetic foot amputation/ulcer healing	Against	Low	Weak	Low
Psychological support ^13^	Prevention of diabetic foot ulcers	In favor	Moderate	Conditional	High
Mindfulness ^18^	Glycemic control	In favor	Low	Weak	High
Ayurveda ^14^	Glycemic control	In favor	Moderate	Conditional	High
Self-care education ^13^	Prevention of diabetic foot ulcers	In favor	Moderate	Conditional	High
Low-impact exercises ^18^	Glycemic control	In favor	Low	Weak	High
Foot- and ankle-focused exercises ^13^	Prevention of diabetic foot ulcers	In favor	Moderate	Conditional	High
Essential oils ^17^	Diabetic foot amputation	In favor (as additional support)	Low	Weak	Low
Ozone therapy ^15^^,^^16^^,^^19^	Diabetic foot amputation/ulcer healing	Against	Low	Weak	High

## Discussion 

This study identified a limited number of clinical guidelines addressing the use of integrative and complementary health practices (ICHP) in diabetes management. The analyzed documents varied in methodological rigor, ranging from well-structured evaluations to recommendations issued without a clear description of criteria. Recommendations were also inconsistent: practices such as Ayurveda, exercise, and mindfulness were suggested in some contexts, whereas biological interventions, such as apitherapy and ozone therapy, were largely discouraged. This combination of scarce evidence and divergent recommendations reflects the fragmentation of the field and underscores the difficulty of establishing solid clinical guidance.

Regarding limitations, the methodological assessment using AGREE II was conducted by one reviewer and verified by another, which may introduce a risk of detection bias. This risk is recognized in rapid reviews, which often employ a single reviewer with independent verification as a pragmatic strategy ^20^. The eligibility process involved typical simplifications of this design, such as excluding gray literature and regional databases, which may have limited the scope of documents identified ^21^. The included guidelines showed heterogeneity in outcomes and populations evaluated, complicating direct comparisons ^12^. To mitigate these limitations, standardized assessment criteria were applied, widely recognized databases were prioritized, and recommendations were grouped by outcome and analyzed qualitatively.

Comparison with previous studies revealed converging findings. For example, complementary therapies such as medicinal herbs and mind-body practices may contribute to glycemic control, although the evidence is of low quality and at high risk of bias ^22^. Regarding the recommendation of complementary practices in guidelines for type 2 diabetes, low frequency and variable methodological quality were identified, reinforcing the need for greater rigor in the development of these documents ^23^. 

The incorporation of practices such as yoga, mindfulness, and music therapy in diabetes care programs has also been described, suggesting potential benefits, albeit with significant methodological limitations ^24^^,^^25^. These findings are consistent with this scenario and indicate that the adoption of ICHP in diabetes management still relies on fragmented evidence.

From a biological perspective, some mechanisms may help explain the observed outcomes. Traditionally used phytotherapeutic drugs in Ayurveda include compounds that may stimulate insulin release, increase insulin sensitivity, and reduce intestinal glucose absorption ^22^^,^^26^. Mind-body interventions, such as yoga and mindfulness, have been associated with reductions in stress and inflammation and with increased adherence to self-care. These effects, in turn, may be associated with improvements in metabolic parameters in individuals with diabetes ^22^. 

Biological therapies such as apitherapy and ozone therapy, on the other hand, lack plausible mechanisms and robust experimental evidence, which is why current recommendations tend to discourage their use. In animal models, practices such as acupuncture have been investigated for potential effects on insulin sensitivity and energy metabolism; however, these findings remain preliminary and do not permit direct extrapolation to clinical recommendations ^27^^,^^28^.

From a public policy perspective, the findings of this rapid review have relevant implications. Within the framework of the National Policy on Integrative and Complementary Practices (PNPIC), these therapeutic resources should be integrated into primary care, taking into account available evidence and the health system's demands ^1^. At the global level, rapid reviews are recognized as instruments that provide timely evidence to support health decision-making ^29^. 

Practices such as self-care programs, exercise, and mindfulness can be considered complementary support, as they may have a positive, though modest, impact on metabolic parameters ^30^^,^^31^. On the other hand, therapies such as ozone therapy and apitherapy lack sufficient scientific evidence to support broad adoption, underscoring the need for caution. ^32^^,^^33^.

This rapid review identified a few guidelines on ICHP in diabetes management, with methodological variability and frequently inconsistent recommendations. Although some practices show initial evidence of benefit, such as self-care programs, exercise, and mindfulness, robust support is still required for widespread implementation in public policies or clinical practice. 

This synthesis underscores the usefulness of rapid reviews for providing timely evidence to support decisions. However, it highlights the need for more comprehensive systematic reviews and guidelines developed with greater methodological rigor that provide more consistent and reliable recommendations for incorporating ICHP into the care of people with diabetes.

## Data Availability

No databases and/or analytical codes were created in this research, beyond the illustrations already available in the manuscript.
