# Erratum: Integrative and complementary practices in diabetes management: a rapid review of clinical guidelines

**DOI:** 10.1590/S2237-96222026v35e20263003.en

**Published:** 2026-07-30

In the article: *Silva MT, Amado DM, Rocha PRS, Barreto JOM. Integrative and complementary practices in diabetes management: a rapid review of clinical guidelines*. Epidemiol Serv Saúde. 2026;35:e20250602. 

https://doi.org/10.1590/S2237-96222026v35e20250602.en, on page 1

Original text:

“Marcos Tolentino Silva’’

Corrected text:

“Marcus Tolentino Silva’’

