# Supplementary material for: Integrative and complementary practices in diabetes management: a rapid review of clinical guidelines
Source: Epidemiol Serv Saude. 2026 Mar 4;35:e20250602. doi: 10.1590/S2237-96222026v35e20250602.en (PMC13686405; doi:10.1590/S2237-96222026v35e20250602.en)
Supplement: Translation

# Translation: Práticas integrativas e complementares no manejo do diabetes: revisão rápida de diretrizes clínicas

**DOI:** 10.1590/S2237-96222026v35e20250602.pt

**Authors:** Marcus Tolentino Silva, Daniel Miele Amado, Paulo Roberto Sousa Rocha, Jorge Otávio Maia Barreto

**Affiliations:** ¹ Universidade de Brasília, Departamento de Saúde Coletiva, Brasília, DF, Brazil Universidade de Brasília Departamento de Saúde Coletiva Brasília DF Brazil; ² Ministério da Saúde, Núcleo Técnico de Gestão da Política Nacional de Práticas Integrativas e Complementares em Saúde no Sistema Único de Saúde, Brasília, DF, Brazil Ministério da Saúde Núcleo Técnico de Gestão da Política Nacional de Práticas Integrativas e Complementares em Saúde no Sistema Único de Saúde Brasília DF Brazil; ³ Fundação Oswaldo Cruz, Escola de Governo, Brasília, DF, Brazil Fundação Oswaldo Cruz Escola de Governo Brasília DF Brazil

**Keywords:** Guia de Prática Clínica, Terapias Complementares, Diabetes Mellitus, Política Informada por Evidência, Revisão Rápida

## Abstract

**Objetivo:**

Mapear diretrizes clínicas que utilizaram o sistema Classificação de Recomendações, Avaliação, Desenvolvimento e Avaliação (*Grading of Recommendations, Assessment, Development and Evaluation*, GRADE), para fazer recomendações sobre práticas integrativas e complementares em saúde (PICS) no manejo do diabetes.

**Métodos:**

A busca foi realizada em Medline/PubMed, Embase e Scopus em 24 de outubro de 2024, sem limite de data. Incluíram-se diretrizes que aplicaram o GRADE. A triagem de títulos e resumos foi duplicada e independente. Um revisor realizou a leitura completa e extração dos dados, e outro fez a verificação. A qualidade metodológica foi avaliada pelo Instrumento de Avaliação de Diretrizes para Pesquisa e Avaliação II (*Appraisal of Guidelines for Research and Evaluation II*, AGREE II), domínio rigor do desenvolvimento. Uma síntese narrativa foi organizada por desfecho.

**Resultados:**

Das sete diretrizes incluídas, cinco apresentaram alta confiança metodológica, uma moderada e uma baixa. As recomendações foram favoráveis para apoio psicológico, educação em autocuidado, exercícios direcionados ao pé e tornozelo, exercícios de baixo impacto, atenção plena, ayurveda e óleos essenciais como suporte adicional; e contrárias para apiterapia e ozonioterapia. Observou-se heterogeneidade nos desfechos e variação na consistência das recomendações.

**Conclusão:**

Faltam diretrizes sobre PICS no manejo do diabetes, com variabilidade metodológica e recomendações inconsistentes. Novas diretrizes, com maior rigor e transparência, podem apoiar decisões sobre a incorporação de PICS no Sistema Único de Saúde. Entre as práticas avaliadas, exercícios, atenção plena e componentes de autocuidado apresentaram evidências mais promissoras, podendo ser priorizadas na atenção primária no cuidado do diabetes.

## Introdução

As práticas integrativas e complementares em saúde (PICS) englobam intervenções de caráter mente-corpo, terapias biológicas e sistemas médicos tradicionais. No Brasil, sua regulamentação está formalizada pela Política Nacional de Práticas Integrativas e Complementares, em vigor desde 2006 no Sistema Único de Saúde (SUS), que prevê a oferta de modalidades como acupuntura, fitoterapia, meditação e yoga ^1^.

O diabetes representa uma das condições crônicas de maior impacto global, com 529 milhões de pessoas afetadas em 2021 e projeção de mais de 1,31 bilhão de casos em 2050 ^2^. Estima-se que o tipo 2 tenha respondido por 97% dos óbitos por diabetes, entre 1990 e 2021, o que reflete o peso da doença em mortalidade e incapacidade ^3^. No Brasil, dados de 2019 indicaram prevalência autorrelatada de 8% (intervalo de confiança de 95% 7,4; 8,0) entre adultos, o que confirmou a magnitude do problema também em âmbito nacional ^4^.

A Classificação de Recomendações, Avaliação, Desenvolvimento e Avaliação (Grading of Recommendations, Assessment, Development and Evaluation, GRADE) é amplamente recomendada como sistema padrão para avaliar a evidência em saúde ^5^^,^^6^, mas sua adoção em diretrizes clínicas ainda é inconsistente. O método classifica a qualidade da evidência (alta, moderada, baixa ou muito baixa) e a força das recomendações (fraca, condicional ou forte), oferecendo maior transparência ao processo de formulação de recomendações clínicas. Na Austrália, no período 2011-2018, entre 240 documentos de diretrizes avaliados, apenas 15 aplicaram o GRADE, de forma heterogênea ^7^. Essa variabilidade reforça a importância de analisar criticamente como as recomendações sobre PICS vêm sendo formuladas.

Embora o manejo convencional do diabetes seja bem estabelecido, desafios persistentes relacionados ao autocuidado, à adesão ao tratamento e ao impacto psicossocial da doença têm motivado o crescente interesse pelas PICS e sua expansão no SUS pela Política Nacional de Práticas Integrativas e Complementares. O escalonamento dessas intervenções requer diretrizes clínicas consistentes, capazes de orientar decisões mais bem informadas sobre benefícios, riscos e incertezas dessas práticas no cuidado de pessoas com diabetes. Assim, justifica-se a necessidade de analisar as recomendações existentes, especialmente à luz do GRADE, como parte do esforço para apoiar gestores e profissionais nas suas decisões e para identificar as lacunas que demandem pesquisas mais robustas.

Esta revisão rápida teve como objetivo mapear diretrizes clínicas que utilizaram o sistema Classificação de Recomendações, Avaliação, Desenvolvimento e Avaliação (Grading of Recommendations, Assessment, Development and Evaluation, GRADE), para fazer recomendações sobre práticas integrativas e complementares em saúde (PICS) no manejo do diabetes.

## Métodos

Protocolo e registro

Esta revisão rápida foi conduzida de acordo com diretrizes metodológicas desenvolvidas a partir de um painel Delphi com especialistas em avaliação de tecnologias em saúde ^8^. Neste caso, o método foi escolhido para atender à necessidade de fornecer evidências sobre PICS no SUS em tempo oportuno. As simplificações incluíram formulação precisa da pergunta de pesquisa, foco em estudos secundários, uso de ferramentas específicas para avaliação crítica e extração de dados por um revisor com verificação por outro. 

A pergunta norteadora foi estruturada com base no acrônimo PICO (population, intervention, comparison, outcome), conforme a seguir.

População (population): pessoas com diabetes. 

Intervenção (intervention): práticas integrativas e complementares em saúde. 

Comparação (comparison): não aplicável. 

Desfechos (outcome): controle glicêmico, cicatrização de feridas e prevenção de úlceras e amputações. 

O relato adaptou as recomendações do checklist Principais itens para relatar Revisões sistemáticas e Meta-análises (Preferred Reporting Items for Systematic Reviews and Meta-Analyses, PRISMA 2020 ^9^. O protocolo foi registrado previamente na plataforma Open Science Framework (disponível em: https://osf.io/q3sbw).

Critérios de elegibilidade

Foram incluídas diretrizes clínicas que utilizaram o sistema GRADE para classificar a qualidade da evidência e a força das recomendações, de forma a assegurar comparabilidade entre documentos e transparência na avaliação das recomendações ^10^. As diretrizes deveriam apresentar recomendações explícitas sobre o uso de PICS em pessoas com diabetes tipo 1, tipo 2 ou complicações associadas. Não houve restrição de idioma, país e período de publicação.

As práticas contempladas foram aquelas avaliadas diretamente nas diretrizes incluídas, abrangendo intervenções voltadas não apenas ao tratamento da doença, mas também à prevenção de complicações, promoção do autocuidado e apoio psicossocial. Buscou-se, na medida do possível, alinhamento com as práticas atualmente vigentes no SUS. Foram excluídas diretrizes que não contemplaram PICS, documentos que não utilizaram o sistema GRADE, consensos de especialistas não embasados em revisão da literatura e diretrizes sem metodologia explícita de busca e de síntese de evidências.

Fontes de informação

Foram utilizadas as bases Medline (via PubMed, Medical Literature Analysis and Retrieval System Online), Embase (Excerpta Medica Database) e Scopus (Elsevier abstract and citation database). Não foram realizadas buscas em portais especializados em diretrizes, como o Guideline International Network ou o National Institute for Health and Care Excellence; bases regionais (como as asiáticas), nem em literatura cinzenta, pois esta revisão foi conduzida em formato rápido, o que exigiu simplificar a etapa de busca ^11^.

Estratégia de busca

A estratégia de busca foi elaborada pelo autor MTS e validada por JOMB. A busca foi realizada em 24 de outubro de 2024 e não se delimitou idioma, país e período de publicação. Foram combinados termos relacionados a “diabetes”, “diretrizes clínicas” e “sistema GRADE”, com ajustes conforme a especificidade de cada base de dados.

Foram empregados operadores booleanos e descritores controlados (MeSH e Emtree), visando equilibrar sensibilidade e especificidade. Embora a pergunta de pesquisa tenha sido estruturada segundo os elementos do acrônimo em inglês PICO, optou-se por não incluir intervenções e desfechos na estratégia, a fim de ampliar a sensibilidade e evitar perda de diretrizes potencialmente relevantes (Tabela 1). 

**Tabela 1:** Estratégia de busca nas fontes de informação selecionadas e resultados obtidos em 24 de outubro de 2024

Fonte	Termos de busca	Resultados
Medline (via PubMed)	#1 “diabetes mellitus”[Mesh] OR (diabetes mellitus) OR (hyperglycemia AND glucose AND intolerance) #2 (guidelines as topic[MeSH:noexp] OR practice guidelines as topic[MeSH:noexp] OR Health Planning Guidelines[MeSH:noexp] OR practice guideline[MeSH:noexp] OR clinical protocols[MeSH:noexp] OR Consensus[MeSH:noexp] OR “Consensus Development Conference”[PTYP] OR “Consensus Development Conference, NIH”[PTYP] OR “Consensus Development Conferences as Topic”[MeSH:noexp] OR “Consensus Development Conferences, NIH as Topic”[MeSH:noexp] OR critical pathway[MeSH:noexp] OR (clinical[TIAB] AND pathway[TIAB]) OR (clinical[TIAB] AND pathways[TIAB]) OR (practice[TIAB] AND parameter[TIAB]) OR (practice[TIAB] AND parameters[TIAB]) OR algorithms[MeSH:noexp] OR care pathway[TIAB] OR care pathways[TIAB] OR guidance[TIAB] OR guideline*[TI]) #3 “GRADE system”[tiab] OR “GRADE methods”[tiab] OR “GRADE approaches”[tiab] OR “GRADE approach”[tiab] OR “Grading of Recommendations, Assessment, Development and Evaluation”[tiab] OR (“GRADE”[tiab] AND (“low”[tiab] OR “moderate”[tiab]OR “high”[tiab] OR “evidence”[tiab] OR “strong”[tiab] OR “weak”[tiab] “recommendation”[tiab])) #4 #1 AND #2 AND #3	119
Embase	1 ‘diabetes mellitus’/exp OR ‘diabetes mellitus’ OR ((‘hyperglycemia’/exp OR ‘hyperglycemia’) AND (‘glucose’/exp OR ‘glucose’) AND (‘intolerance’/exp OR ‘intolerance’)) #2 ‘practice guideline’/exp OR ‘practice guideline’ #3 ‘GRADE approach’/exp OR ‘GRADE approach’ #4 #1 AND #2 AND #3	104
SCOPUS	TITLE-ABS-KEY(“diabetes mellitus”) OR TITLE-ABS-KEY(“hyperglycemia” AND “glucose” AND “intolerance”)) AND (TITLE-ABS-KEY(“GRADE system” OR “GRADE methods” OR “GRADE approaches” OR “GRADE approach” OR “Grading of Recommendations, Assessment, Development and Evaluation”))	309

Processo de seleção

A seleção dos estudos ocorreu em duas etapas. Inicialmente, dois revisores independentes (MTS e JOMB) examinaram títulos e resumos, aplicando os critérios de elegibilidade. Em seguida, os textos completos dos registros potencialmente relevantes foram avaliados por MTS, com verificação por JOMB. 

As divergências foram resolvidas por consenso. A remoção de duplicatas e a organização da triagem foram realizadas no EndNote X8.2 (Clarivate Analytics). Durante a redação do protocolo da resposta rápida, conduziu-se um teste-piloto, que auxiliou na identificação de referências sentinela e permitiu ajustes e correções na estratégia de busca.

Extração de dados

A extração de dados foi realizada por MTS, utilizando planilha padronizada no Microsoft Word, e verificada de forma independente por JOMB. Em caso de discordância, as decisões foram tomadas por consenso entre ambos os revisores. Quando determinada variável não estava claramente descrita nos documentos, utilizou-se a expressão “não informado” nas tabelas, sem inserção de dados adicionais ou contato com os autores das diretrizes, em consonância com o delineamento de revisão rápida.

Foram extraídas as seguintes variáveis: autor e ano da diretriz, contexto (país ou abrangência), condição-alvo (diabetes tipo 1, tipo 2 ou complicações associadas), práticas integrativas contempladas, desfechos avaliados, recomendações formuladas, qualidade da evidência (muito baixa, baixa, moderada ou alta, segundo o sistema GRADE) e força da recomendação (fraca, condicional ou forte, conforme o GRADE).

Avaliação da qualidade metodológica

A qualidade metodológica das diretrizes foi avaliada com base no domínio 3 (rigor do desenvolvimento) do Instrumento de Avaliação de Diretrizes para Pesquisa e Avaliação II (Appraisal of Guidelines for Research and Evaluation II, AGREE II) ^12^. Optou-se por utilizar apenas os oito itens desse domínio, em vez dos 28 itens completos da ferramenta, considerando-se o delineamento de revisão rápida e o foco na robustez da formulação das recomendações.

Para cada item do domínio, atribuiu-se o julgamento: sim (critério atendido), parcial (atendimento incompleto) ou não (critério não atendido). Os itens avaliados foram: busca sistemática de evidências, critérios de seleção explícitos, descrição de forças e limitações, método de formulação das recomendações, consideração de benefícios e riscos, relação transparente entre evidências e recomendações, revisão externa e previsão de atualização. A classificação final da confiança metodológica das diretrizes foi operacionalizada como: alta (todos os oito itens atendidos), moderada (alguns itens atendidos parcialmente) e baixa (múltiplas respostas “não”).

Síntese dos dados

As informações extraídas foram organizadas em síntese narrativa e apresentadas em tabelas estruturadas por desfecho clínico e pelas práticas integrativas avaliadas. Essa apresentação buscou facilitar a visualização conjunta da qualidade metodológica das diretrizes, da consistência das recomendações e da força com que foram emitidas.

A consistência das recomendações foi avaliada pela comparação entre as diretrizes incluídas para cada prática e desfecho, permitindo identificar convergências, divergências e ausência de recomendações. A força das recomendações foi descrita segundo o sistema GRADE, podendo ser classificada como forte, fraca ou condicional. A combinação das recomendações ocorreu de forma qualitativa e buscou o equilíbrio a partir da análise crítica do conjunto de evidências.

## Resultados 

A busca realizada identificou 532 registros. Após a remoção de 151 duplicatas, restaram 381 registros para triagem de títulos e resumos. Destes, 326 foram excluídos por não atenderem aos critérios de elegibilidade. Foram avaliados 55 textos completos, com exclusão de 48 diretrizes que não contemplaram PICS. A relação detalhada dessas diretrizes está disponível como material suplementar (disponível em: https://osf.io/q3sbw). Ao final, 7 diretrizes clínicas ^13^^-^^19^ foram incluídas na síntese (Figura 1).

**Figura 1:** Fluxo de identificação das diretrizes clínicas

**Tabela 2:** Características das diretrizes clínicas incluídas sobre práticas integrativas e complementares em saúde no manejo do diabetes (n=7)

Autor, ano	Contexto da diretriz	Condição-alvo	Práticas avaliadas	Desfechos
Bus, 2023 ^13^	Global	Úlceras do pé diabético	Exercícios direcionados ao pé e tornozelo, educação em autocuidado, apoio psicológico	Prevenção de úlceras do pé diabético
Chattopadhyay, 2023 ^14^	Índia	Diabetes tipo 2	Ayurveda	Controle glicêmico
Chen, 2022 ^15^	Austrália	Úlceras do pé diabético	Ozonioterapia	Cicatrização de úlceras do pé diabético
Chen, 2024 ^16^	Global	Úlceras do pé diabético	Ozonioterapia	Cicatrização de úlceras do pé diabético
Commons, 2022 ^17^	Austrália	Infecções associadas ao diabetes	Apiterapia, óleos essenciais	Incidência de amputações
Conlin, 2024 ^18^	Estados Unidos	Diabetes tipo 2	Exercícios de baixo impacto, atenção plena	Controle glicêmico
Lazzarini, 2023 ^19^	Austrália	Úlceras do pé diabético	Apiterapia, ozonioterapia	Incidência de amputações

**Tabela 3:** Avaliação crítica das diretrizes clínicas incluídas sobre diabetes segundo critérios do Instrumento de Avaliação de Diretrizes para Pesquisa e Avaliação II (Appraisal of Guidelines for Research and Evaluation II, AGREE II)

Autor, ano	Busca sistemática	Critérios de seleção	Forças/limitações descritas	Formulações das recomendações	Benefícios e riscos considerados	Relação entre recomendações e evidências	Revisão externa	Atualização prevista	Confiança da diretriz
Bus, 2023 ^13^	Sim	Sim	Sim	Sim	Sim	Sim	Sim	Sim	Alta
Chattopadhyay, 2023 ^14^	Sim	Sim	Sim	Sim	Sim	Sim	Sim	Sim	Alta
Chen, 2022 ^15^	Parcial	Parcial	Parcial	Parcial	Sim	Parcial	Não	Não	Moderada
Chen, 2024 ^16^	Sim	Sim	Sim	Sim	Sim	Sim	Sim	Sim	Alta
Commons, 2022 ^17^	Não	Não	Não	Não	Não	Não	Não	Não	Baixa
Conlin, 2024 ^18^	Sim	Sim	Sim	Sim	Sim	Sim	Sim	Sim	Alta
Lazzarini, 2023 ^19^	Sim	Sim	Sim	Sim	Sim	Sim	Sim	Sim	Alta

**Tabela 4:** Síntese das recomendações de uso de práticas integrativas e complementares em saúde em diretrizes clínicas sobre diabetes

Prática avaliada (referência)	Desfecho	Direcionamento	Qualidade da evidência	Força da recomendação	Consistência da recomendação
Apiterapia ^17^^,^^19^	Amputação do pé diabético/cicatrização de úlceras	Contra	Baixa	Fraca	Baixa
Apoio psicológico ^13^	Prevenção de úlceras do pé diabético	A favor	Moderada	Condicional	Alta
Atenção plena ^18^	Controle glicêmico	A favor	Baixa	Fraca	Alta
Ayurveda ^14^	Controle glicêmico	A favor	Moderada	Condicional	Alta
Educação em autocuidado ^13^	Prevenção de úlceras do pé diabético	A favor	Moderada	Condicional	Alta
Exercícios de baixo impacto ^18^	Controle glicêmico	A favor	Baixa	Fraca	Alta
Exercícios direcionados ao pé e tornozelo ^13^	Prevenção de úlceras do pé diabético	A favor	Moderada	Condicional	Alta
Óleos essenciais ^17^	Amputação do pé diabético	A favor (como suporte adicional)	Baixa	Fraca	Baixa
Ozonioterapia ^15^^,^^16^^,^^19^	Amputação do pé diabético/cicatrização de úlceras	Contra	Baixa	Fraca	Alta

## Discussão

Este estudo identificou um número reduzido de diretrizes clínicas que abordam o uso de práticas integrativas e complementares em saúde no manejo do diabetes. Os documentos analisados variaram quanto ao rigor metodológico, indo de avaliações bem estruturadas até recomendações emitidas sem descrição clara de critérios. As recomendações também foram inconsistentes: práticas como ayurveda, exercícios e atenção plena foram sugeridas em alguns contextos analisados, enquanto intervenções biológicas, como apiterapia e ozonioterapia, foram majoritariamente desaconselhadas. Essa combinação de evidências escassas e recomendações divergentes reflete a fragmentação do campo e reforça a dificuldade de estabelecer orientações clínicas sólidas.

Quanto às limitações, a avaliação metodológica pelo AGREE II foi conduzida por um revisor e verificada por outro, o que pode introduzir risco de viés de detecção. Esse risco é reconhecido em revisões rápidas, que frequentemente utilizam um único revisor com checagem independente como estratégia pragmática ^20^. O processo de elegibilidade envolveu simplificações típicas desse delineamento, como não incluir literatura cinzenta ou bases regionais, o que pode ter restringido a abrangência dos documentos identificados ^21^. As diretrizes incluídas mostraram heterogeneidade nos desfechos e nas populações avaliadas, dificultando comparações diretas ^12^. Para mitigar essas limitações, foram utilizados critérios padronizados de avaliação, priorizadas bases amplamente reconhecidas e agrupadas recomendações por desfecho, analisando-as qualitativamente.

Ao comparar com estudos prévios, observou-se convergência de achados. Por exemplo, terapias complementares como ervas medicinais e práticas mente-corpo podem contribuir para o controle glicêmico, mas com evidência de baixa qualidade e alto risco de viés ^22^. Para a recomendação de práticas complementares em diretrizes para diabetes tipo 2, foram identificadas baixa frequência e qualidade metodológica variável, reforçando a necessidade de maior rigor no desenvolvimento desses documentos ^23^. 

Também foi descrita a incorporação de práticas como yoga, atenção plena e musicoterapia em programas de cuidado a pessoas com diabetes, sugerindo benefícios potenciais, embora com limitações metodológicas relevantes ^24^^,^^25^. Estes achados são consistentes com esse panorama e indicam que a adoção de PICS no manejo do diabetes ainda se apoia em evidências fragmentadas.

Sob a perspectiva biológica, alguns mecanismos podem ajudar a compreender os resultados. Fitoterápicos tradicionalmente empregados, como parte do ayurveda, incluem compostos com potencial para estimular a liberação de insulina, aumentar a sensibilidade do organismo à sua ação e reduzir a absorção intestinal de glicose ^22^^,^^26^. Intervenções mente-corpo, como yoga e atenção plena, têm sido associadas à redução do estresse e da inflamação, bem como ao aumento da adesão ao autocuidado. Tais efeitos, por sua vez, podem estar relacionados à melhora de parâmetros metabólicos em pessoas com diabetes ^22^. 

As terapias biológicas como apiterapia e ozonioterapia, por outro lado, carecem de mecanismos plausíveis e de evidências experimentais robustas, razão pela qual as recomendações atuais tendem a desaconselhar seu uso. Em modelos animais, práticas como a acupuntura têm sido investigadas quanto a possíveis efeitos sobre a sensibilidade à insulina e o metabolismo energético; no entanto, esses achados permanecem preliminares e não permitem extrapolação direta para recomendações clínicas ^27^^,^^28^.

Do ponto de vista das políticas públicas, os achados desta revisão rápida têm implicações relevantes. No âmbito da Política Nacional de Práticas Integrativas e Complementares, a incorporação desses recursos terapêuticos deve ocorrer de forma integrada à atenção primária, considerando as evidências disponíveis e as demandas do sistema de saúde ^1^. Em nível global, revisões rápidas são reconhecidas como instrumentos que permitem fornecer subsídios em tempo oportuno para decisões em saúde ^29^. 

Práticas como programas de autocuidado, exercícios e atenção plena podem ser consideradas como apoio complementar, já que podem representar impacto positivo, ainda que modesto, sobre parâmetros metabólicos ^30^^,^^31^. Por outro lado, terapias como ozonioterapia e apiterapia permanecem sem respaldo científico suficiente para adoção ampla, reforçando a necessidade de cautela ^32^^,^^33^.

Esta revisão rápida identificou poucas diretrizes sobre PICS no manejo do diabetes, com variabilidade metodológica e recomendações frequentemente inconsistentes. Embora algumas práticas apresentem evidências iniciais de benefício, como programas de autocuidado, exercícios e atenção plena, ainda se faz necessário suporte robusto para aplicação ampla em políticas públicas ou prática clínica. 

Esta síntese reforça a utilidade das revisões rápidas para subsidiar decisões em tempo oportuno. Contudo, aponta para a necessidade de revisões sistemáticas mais abrangentes e de diretrizes elaboradas com maior rigor metodológico, capazes de oferecer recomendações mais consistentes e seguras para incorporar as PICS no cuidado de pessoas com diabetes.

As diretrizes incluídas foram publicadas entre 2022 e 2024 e abrangiam diferentes iniciativas globais e contextos, como os da Índia, da Austrália e dos Estados Unidos. As diretrizes incluídas abordaram o manejo do diabetes tipo 2 e de complicações como úlceras do pé diabético e infecções associadas ao diabetes (Tabela 2). Entre as práticas avaliadas, destacaram-se exercícios direcionados ao pé e tornozelo, educação em autocuidado, apoio psicológico, atenção plena, ayurveda, óleos essenciais, exercícios de baixo impacto, apiterapia e ozonioterapia. Os desfechos analisados incluíram controle glicêmico, prevenção e cicatrização de úlceras e incidência de amputações.

A avaliação metodológica pelo instrumento AGREE II mostrou 5 diretrizes com alta confiança, 1 moderada e 1 baixa (Tabela 3). Aquelas classificadas como de alta confiança atenderam, de forma consistente, a critérios como busca sistemática, descrição dos métodos de seleção, clareza na formulação das recomendações e relação explícita entre recomendações e evidências. A diretriz de confiança moderada atendeu parcialmente a esses critérios, enquanto a de menor qualidade não contemplou nenhum dos itens avaliados. As classificações finais foram atribuídas conforme a proporção de critérios atendidos.

As recomendações das diretrizes apresentaram variabilidade (Tabela 4). Apoio psicológico, educação em autocuidado e exercícios direcionados ao pé e tornozelo foram condicionalmente recomendados para prevenção de úlceras do pé diabético (evidência moderada; consistência alta). O ayurveda foi recomendado de forma condicional como complemento no controle glicêmico (evidência moderada; consistência alta). Exercícios de baixo impacto e atenção plena foram recomendados de forma fraca para controle glicêmico (evidência baixa; consistência alta). Óleos essenciais foram sugeridos como suporte adicional no manejo do pé diabético (evidência baixa; consistência baixa). Apiterapia e ozonioterapia não foram recomendadas para cicatrização de úlceras e prevenção de amputações (evidência baixa; consistência baixa a alta).

## Data Availability

Não foram criados banco de dados e/ou códigos de análise nesta pesquisa, além das ilustrações já disponíveis no manuscrito.
