# Supplementary material for: Erratum: Integrative and complementary practices in diabetes management: a rapid review of clinical guidelines
Source: Epidemiol Serv Saude. 2026 Jul 30;35:e20263003. doi: 10.1590/S2237-96222026v35e20263003.en (PMC13686292; doi:10.1590/S2237-96222026v35e20263003.en)
Supplement: Translation

# Translation: Errata: Práticas integrativas e complementares no manejo do diabetes: revisão rápida de diretrizes clínicas

**DOI:** 10.1590/S2237-96222026v35e20263003.pt

No artigo: *Silva MT, Amado DM, Rocha PRS, Barreto JOM. Práticas integrativas e complementares no manejo do diabetes: revisão rápida de diretrizes clínicas*. Epidemiol Serv Saúde. 2026;35:e20250602. 

https://doi.org/10.1590/S2237-96222026v35e20250602.pt, na página 1

Onde se lê:

“Marcos Tolentino Silva’’

Leia-se:

“Marcus Tolentino Silva’’
